# Risk factors associated with symptomatic cholelithiasis in Taiwan: a population-based study

**DOI:** 10.1186/1471-230X-11-111

**Published:** 2011-10-17

**Authors:** Shih-Chang Hung, Kuan-Fu Liao, Shih-Wei Lai, Chia-Ing Li, Wen-Chi Chen

**Affiliations:** 1 Department of Emergency Medicine, Nantou Hospital, Nantou, 540, Taiwan; 2 Department of Internal Medicine, Taichung Tzu Chi General Hospital, Taichung, 427, Taiwan; 3 School of Medicine, Tzu Chi University, Hualien, 970, Taiwan; 4 School of Medicine, China Medical University, Taichung, 404, Taiwan; 5 Department of Family Medicine, China Medical University Hospital, Taichung, 404, Taiwan; 6 Department of Medical Research, China Medical University Hospital, Taichung, 404, Taiwan; 7 School of Chinese Medicine, China Medical University, Taichung, 404, Taiwan; 8 Department of Urology, China Medical University Hospital, Taichung, 404, Taiwan; 9 Department of Public Health, China Medical University, Taichung, 404, Taiwan

**Keywords:** cholelithiasis, hepatitis B, hepatitis C, hyperlipidemia, menopause, obesity

## Abstract

**Background:**

Cholelithiasis has become a major health problem in Taiwan. The predominant type of gallstone found in Asian populations differs from that in the West, indicating possible differences in the etiology and risk factors for cholelithiasis. The aim of this study is to investigate the risk factors for cholelithiasis using data representative of the general population.

**Methods:**

We performed a population-based, case-control study in which we analyzed medical data for 3725 patients newly diagnosed with cholelithiasis and 11175 gender- and age-matched controls with no history of cholelithiasis, using information obtained from the 2005 Registry for Beneficiaries of the National Health Insurance Research Database. Coexisting medical conditions were included in the analysis. Relative risks were estimated by adjusted odds ratio (OR) and 95% confidence interval (CI) using a multivariate logistic regression analysis.

**Results:**

After controlling for the other covariates, multivariate logistic regression analysis identified the following as risk factors for cholelithiasis (in descending order of contribution): Among all patients - hepatitis C (OR = 2.78), cirrhosis (OR = 2.47), hepatitis B (OR = 2.00), obesity (OR = 1.89), and hyperlipidemia (OR = 1.54); Among women - hepatitis C (OR = 3.05), cirrhosis (OR = 1.92), obesity (OR = 1.91), menopause (OR = 1.61), hepatitis B (OR = 1.54), and hyperlipidemia (OR = 1.49). Diabetes mellitus appeared to have a marked influence on the development of cholelithiasis but was not identified as a significant independent risk factor for cholelithiasis.

**Conclusions:**

The risk factors for cholelithiasis were obesity, hyperlipidemia, hepatitis B infection, hepatitis C infection, and cirrhosis in both genders, and menopause in females. Despite differences in the predominate type of gallstone in Asian versus Western populations, we identified no unique risk factors among the population of Taiwan.

## Background

Cholelithiasis (the presence of one or more gallstones in the bladder) represents a significant burden for healthcare systems worldwide [1] and is one of the most common disorders among patients presenting to emergency rooms with abdominal discomfort--e.g., epigastric pain, nausea, vomiting, loss of appetite [2]. Ethnicity and family traits are recognized as contributing factor [3]. Cholelithiasis is known to affect 60-70% of Native Americans and a proportionately smaller number individuals of mixed Hispanic/Native American background [4]. The incidence is at least 10% among white adults in Western countries [5], and even lower in African Americans and East Asians [4]. Risk factors associated with cholelithiasis in the West include gender (F > M), age, obesity, metabolic syndrome, rapid appetite loss, hepatitis C, cirrhosis, and high caloric intake [4,6].

Cholelithiasis is also on the rise and becoming a major health problem in Taiwan [7,8], with some estimates indicating an increase in prevalence from 4.3% in 1989 to as high as 10.7% in 1995 [8]. In the West, 80-90% of gallstones are cholesterol stones [1], whereas the majority of gallstones found in Asian populations are pigment stones (high-residue pigment, low-residue pigment, and muddy pigment types), derived largely from salts or polymers of bilirubinate [9-11].

In order to establish effective preventative medical strategies for the prevention and detection of cholelithiasis in Taiwan, it will be important to identify and understand risk factors that may be unique in the population. Differences in the prevalent type of gallstone indicate a potential differences in the etiology of cholelithiasis in Taiwan versus the West. A small number of studies have explored risk factors for cholelithiasis in the Taiwanese population [8,12-15]. Chen et al. [12] studied individuals from a rural village in Taiwan and found an overall prevalence of gallstone disease of around 5%. They identified age and fatty liver to be significant risk factors in both sexes, with no overall link to gender. Liu et al. [16] assessed cholelithiasis in healthy, paid volunteers admitted to hospital and identified a prevalence of 5.3%. Cholelithiasis in this study was linked to age, obesity (body mass index [BMI] ≥ 27 kg/m^2^), and type 2 diabetes, but not gender. A few additional studies have identified diastolic blood pressure, elevated alanine aminotransferase levels, and waist circumference as risk factors for cholelithiasis within high risk subgroups of obese individuals [14] and patients with type 2 diabetes [8,15].

The above studies suggest similarities as well as differences between the nature of cholelithiasis in Taiwan and in the West. There is a selection bias associated with each of these studies, however, due to the focus on a particular subgroup within the Taiwanese population, and as a result they may not offer an accurate representation of risk factors for cholelithiasis among the general population. The aim of this study was to perform a case-controlled analysis of medical information from the nation-wide 2005 Registry for Beneficiaries of the National Health Insurance Research Database. While the use of such a database provides access to a wealth of information on the health status of a large number of patients, it also has limitations. For example, the database did not reveal the criteria on which the diagnoses of cholelithiasis were based. Nevertheless, our data offer a better understanding of medical factors contributing to the development of cholelithiasis across the general population of Taiwan.

## Methods

### Study Population

We identified total of 14900 patients from a random sample of the 2005 Registry for Beneficiaries of the National Health Insurance Research Database. This database contains longitudinal insurance records of reimbursement claims for the years 2003 to 2007, which includes the original claim data of 1000000 beneficiaries. This insurance program has covered more than 96% of Taiwan's population and has contracted with 97% of hospitals and clinics in Taiwan, since March 1995 [17]. The database included information regarding date of birth, gender, medical diagnosis, and medical procedures. These data were released for public access and patient identities were not revealed. Information was obtained with the authorization of Bureau of National Health Insurance, Department of Health in Taiwan. This study was exempt from full review by the institution review board.

### Criteria and Definition

We used diagnostic guidelines from the International Classification of Diseases (ICD) 9^th ^Revision [18] to identify individuals newly diagnosed with cholelithiasis (ICD-9-CM 574) who were 20 years of age and above in 2006-2007. Individuals meeting these criteria were selected randomly, without regard to age or gender, for inclusion in the *case group*. Because the database did not provide the diagnostic criteria for cholelithiasis, these patients are more appropriately designated as having symptomatic cholelithiasis, i.e., calculus of the gallbladder without mention of cholecystitis. Each individual in the case group was matched to three individuals of the same age and sex in the *control group*. It should be noted that because the control group was constructed in this manner--i.e., by matching exactly the age and sex of controls to individuals in the case group--the mean ages for the two groups are identical, as are the percentages of males and females in the two groups. Furthermore, because of the large n values, the standard deviations for the case and control groups are identical to within three significant figures (see Table 1). Controls were defined as individuals who had neither been diagnosed with cholelithiasis nor undergone a related medical procedure, such as cholecystectomy (ICD-9-CM 51.22 and 51.23) or common duct exploration for removal of calculus (ICD-9-CM 51.41) between the years 2003 to 2007. The *age *for individuals in the case group was defined as the age of onset of cholelithiasis as determined at the time of diagnosis. Age for individuals in the control group was defined as their age 2007.

**Table 1:** Age and Gender Characteristics of the Study Population^a^

	Symptomatic Cholelithiasis	
	
	Yes (n = 3725)	No (n = 11175)
Age (year)	55.9 ± 15.9	55.9 ± 15.9
Male	1621 (43.5%)	4863 (43.5%)
Female	2104 (56.5%)	6312 (56.5%)

^a ^The average ages and gender percentages for the case and control groups are identical, because individuals in the control group were age- and gender-matched to individuals in the case group.

Medical conditions that were evaluated as possible risk factors for cholelithiasis were obesity (ICD-9-CM 278.00 and 278.01), diabetes mellitus (ICD-9-CM 250), hyperlipidemia (ICD-9-CM 272.0, 272.1, 272.2, 272.3 and 272.4), chronic kidney disease (ICD-9-CM 585, 586, 588.8 and 588.9), hepatitis B infection (ICD-9-CM V02.61, 070.20, 070.22, 070.30 and 070.32), hepatitis C infection (ICD-9-CM V02.62, 070.41, 070.44, 070.51 and 070.54), cirrhosis (ICD-9-CM 571.2, 571.5 and 571.6), and menopause (ICD-9-CM V49.81, 627.2, 627.8 and 627.9). Only a relatively small percentage of individuals, those presenting with acute cholecystitis, would normally undergo emergency cholecystectomy at first presentation. Therefore, we elected to included in this study only individuals who had presented and been diagnosed with cholelithiasis in ambulatory care twice during the period from 2003 to 2005.

### Statistical analysis

Data are presented as the mean ± standard deviation (SD) of *n *determinations; gender was presented as counts with percentages. The associations between potential medical risk factors and cholelithiasis were summarized as odds ratios (OR) with the 95% confidence interval (CI) in the univariate conditional logistic regression models. To investigate the risk factors for cholelithiasis while controlling for the other covariates, those medical conditions meeting the statistical criterion of *P *< 0.1 in the univariate conditional logistic regression models were subjected to multivariate conditional logistic regression analysis. A value of *P *< 0.05 was considered statistically significant in the multivariate analysis. Statistical analyses were performed by SAS software version 9.1 (SAS Institute Inc., Cary, North Carolina) for data analyses with two-sided probability value.

## Results

### Baseline characteristics of the study population

Medical data for 14900 individuals were used in our analysis, including 3725 previously diagnosed with cholelithiasis (symptomatic cholelithiasis; case group) and 11175 with no history of cholelithiasis or medically related procedures (control group). The study was made up of 6484 males (43.5%) and 8416 females (56.5%), and participants ranged in age from 20 to 98 years (mean age = 55.9 ± 15.9 years). It should be noted that because of the study design (see Methods), the age distributions are the same in the case and control groups, as are the mean ages for case and control groups (Table 1).

### Risk factors for cholelithiasis

Univariate analysis identified seven medical conditions as potential risk factors for the development of cholelithiasis: obesity, diabetes mellitus, hyperlipidemia, hepatitis B infection, hepatitis C infection, and cirrhosis were identified in both genders. Menopause was identified as an additional risk factor for cholelithiasis in females (*P *< 0.001) (Table 2).

**Table 2:** Summary for the risk factors of cholelithiasis in univariate conditional logistic regression models

	Cholelithiasis				
	
	Yes (n = 3725)	No (n = 11175)	OR	95% CI	P value
Obesity	30 (0.81%)	42 (0.38%)	2.16	1.35 - 3.45	0.001*
Diabetes mellitus	513 (13.77%)	1 236 (11.06%)	1.31	1.17 - 1.47	< 0.001*
Hyperlipidemia	662 (17.77%)	1 349 (12.07%)	1.62	1.46 - 1.8	< 0.001*
Chronic kidney disease	57 (1.53%)	136 (1.22%)	1.27	0.92 - 1.73	0.142
Hepatitis B	108 (2.90%)	143 (1.28%)	2.31	1.79 - 2.97	< 0.001*
Hepatitis C	82 (2.20%)	76 (0.68%)	3.3	2.41 - 4.52	< 0.001*
Cirrhosis	65 (1.74%)	65 (0.58%)	3.05	2.16 - 4.32	< 0.001*
Menopause^#^	304 (14.45%)	612 (6.70%)	1.65	1.41 - 1.93	< 0.001*

OR: odds ratio; CI: confidence interval. ^#^Calculated in female subjects (2104 cases and 6312 controls). *Indicating the corresponding variable that had significant impact on the occurrence of cholelithiasis. Each OR was calculated by univariate conditional logistic regression

Multivariate conditional logistic regression analysis was used to determine the independence of risk factors associated with cholelithiasis. Individuals with the following conditions were more likely to have cholelithiasis than individuals without the condition: obesity (OR = 1.89 with 95% CI of 1.18-3.04); hyperlipidemia (OR = 1.54 with 95% CI of 1.38-1.72); hepatitis B or C infections (OR = 2.00 with 95% CI of 1.55-2.60 for hepatitis B; OR = 2.78 with 95% CI of 2.01-3.84 for hepatitis C); and cirrhosis (OR = 2.47 with 95% CI of 1.72-3.53). In addition to these risk factors menopause was identified in the multivariate analysis as a risk factor for cholelithiasis in females (OR = 1.61 with 95% CI of 1.38-1.89). Diabetes mellitus was not identified as a conferring a significant risk for the development of cholelithiasis (Table 3).

**Table 3:** Summary for the risk factors of cholelithiasis in Multivariate conditional logistic regression models for all subject and females

	For all subjects (Case, n = 3725; Control, n = 11175)			For female subjects (Case, n = 2104; Control, n = 6312)		
		
	OR	OR (95% CI)	*P *value	OR	OR (95% CI)	*P *value
Obesity	1.89	1.18-3.04	0.008*	1.91	1.07-3.41	0.030*
Diabetes mellitus	1.13	1.00-1.27	0.054	1.09	0.92-1.28	0.323
Hyperlipidemia	1.54	1.38-1.72	< 0.001*	1.49	1.29-1.72	< 0.001*
Hepatitis B	2.00	1.55-2.60	< 0.001*	1.54	1.04-2.28	< 0.001*
Hepatitis C	2.78	2.01-3.84	< 0.001*	3.05	2.01-4.62	< 0.001*
Cirrhosis	2.47	1.72-3.53	< 0.001*	1.92	1.03-3.58	< 0.001*
Menopause^#^	---			1.61	1.38-1.89	< 0.001*

OR: odds ratio; CI: confidence interval. ^#^Calculated in female subjects (2104 cases and 6312 controls). * Indicated the corresponding variable had significant impact on the occurrence of cholelithiasis.

## Discussion and Conclusions

Cholelithiasis in some form-silent gallstones [19], simple biliary colic pain, acute cholecystitis sepsis, and, infrequently, gallstone ileus [20,21]--has accounted for an increasing number of hospital admissions and is a growing healthcare concern in Taiwan [7,8]. Although the growing prevalence of cholelithiasis has been linked informally to an increase in influence of the Western diet, obesity, and sedentary lifestyle, differences in gallstone composition indicate there may be differences as well in the etiology of cholelithiasis in Taiwan compared to the West. Despite differences in the predominate type of gallstone in Asian versus Western populations, we identified no unique risk factors among the population of Taiwan. Using multivariate conditional logistic regression analysis, we identified the following as independent risk factors for cholelithiasis (in descending order of contribution): Among all patients - hepatitis C (OR = 2.78), cirrhosis (OR = 2.47), hepatitis B (OR = 2.00), obesity (OR = 1.89), and hyperlipidemia (OR = 1.54); Among women - hepatitis C (OR = 3.05), cirrhosis (OR = 1.92), obesity (OR = 1.91), menopause (OR = 1.61), hepatitis B (OR = 1.54), and hyperlipidemia (OR = 1.49).

The prevalence of cholelithiasis has been estimated at approximately 4.3 to 14.4%, with a calculated annual incidence of 2.6~3.56% [8,14,15,22,23]. Around one-third of individuals for whom an initial diagnosis of gallstones is asymptomatic or noncritical will undergo a subsequent cholecystectomy for recurrent symptoms or complications [24,25]. Prophylactic surgical treatment for complicated cholecystitis has also been discussed for high risk sub-group individuals [13,19,25]. Identifying the major risk factors for cholelithiasis is an important step in preventing the formation of gallstones and reducing cholelithiasis-related illness.

The majority of studies into risk factors for cholelithiasis have been based on data from Western countries [25,26]. In the Asia-pacific area, especially in Taiwan, research into the incidence, prevalence, and risk factors associated cholelithiasis are usually conducted in a single hospitals, limited areas, or a limited sub-group of individuals [8,14-16]. The introduction of the National Health Insurance database offered a means to analyze data from the general population of Taiwan.

In selecting the medical conditions for analysis, we were constrained by the level of specificity within the database (e.g., data for hyperlipidemia were available, but data for HDL and LDL levels were not provided). We were particularly interested in evaluating medical conditions with particular relevance to the population of Taiwan. Hepatitis B and C were of interest, because their prevalence is much higher in Taiwan than in western countries. Conversely, blood cholesterol level was not evaluated, because the majority of cases of cholelithiasis in Taiwan do not involve cholesterol stones (see below).

Gallstones are generally classified as belonging to one of two types: cholesterol stones, and pigment stones [1]. Pigment stones can be further subdivided into high-residue pigment, low-residue pigment, and muddy pigment stones [9]. Cholesterol stones are formed by the precipitation of cholesterol as solid crystals from supersaturated bile and reflect high levels of hepatic cholesterol secretion. Pigment stones, which are more predominant in the Asian population [9-11], are composed primarily of bilirubinate salts and oxidized tetrapyrrolic derivatives formed by polymerization of unconjugated bilirubin [27]. This type of gallstone is linked to hemolysis, bacterial infection, or liver disease, rather than hormonal factors [1,12]. Others also report an association between pigment gallstones and advancing age and possibly alcoholism [28]. We were unable to directly assess the distribution of gallstone types in this study, because no clinical test reports regarding signs of hyperhemolysis (lower hemoglobin content) were available in the database. Chronic liver disease is a well-recognized risk factor for cholelithiasis [4,23]. Fatty liver [12], hepatitis B [16,22], hepatitis C [6,29], elevated ALT levels [15], and cirrhosis [22,30,31] have all been found to be related to cholelithiasis in different small studies. In Taiwan there is known to be a higher prevalence of viral hepatitis than in other regions [32]. Our study confirms that hepatitis B, hepatitis C, and cirrhosis contribute a significant risk for cholelithiasis. Additional studies to understand the mechanistic relationship between cholelithiasis, virus hepatitis, alcohol-related cirrhosis, viral hepatitis cirrhosis, as well as non-alcoholic fatty liver disease are needed [33].

All of the constituents of metabolic syndrome, with the exception of hypertension, have been reported as independent risk factors for cholelithiasis, including low serum levels of high density lipoprotein [13,34,35], diabetes and glucose intolerance [12,13,16,22,29,34], high BMI [11,14,16,29,36,37], increased waist circumference [15], and obesity [15,34,35,38]. In the present study, we identified obesity and hyperlipidemia as strong risk factors in the general population of Taiwan. Overall, these findings point to a likely benefit of life style and dietary modification as effective measures for the prevention of cholelithiasis [39]. In addition, they support previous observations indicating that medications used to treat dyslipidemia may be of value in the prevention and treatment of cholelithiasis [31,40].

We found a strong association between diabetes and cholelithiasis in univariate analysis, but did not identify diabetes as a significant independent risk factor in multivariate analysis. There remains some controversy in the literature regarding type 2 diabetes as an independent risk factor for cholelithiasis. Previous community-based studies in healthy adults in Taiwan have found diabetes mellitus to be highly associated with the prevalence of gallstone disease [12,16]. In addition, Chen et al. [12] found diabetes to be an independent risk factor for gallstone disease in women, but not in men. Type 2 diabetes has also been reported as an independent risk factor for gallstone disease in women but not men in Western studies [41]. In contrast, other investigators have found no independent link between type 2 diabetes and the development of gallstones. Pacchioni et al. [42] observed that obesity and type 2 diabetes were both more frequent in patients with gallstones than in those without gallstones, and suggested that age and obesity are the main risk factors for gallstones, with type 2 diabetes playing a lesser role in the development of gallstones. Shaffer et al. [4] concluded that while diabetes mellitus may be linked to gallstone formation, the relationship is influenced by co-factors such as age, body mass index, and a family history of gallstone disease. Our results support the idea that type 2 diabetes acts in concert with diet, BMI, and other factors to increase the risk of cholelithiasis.

There remains some controversy about gender as a risk factor for cholelithiasis. While the majority of studies conducted in the West have concluded that females are more likely than males to develop cholelithiasis [4,38,43], several studies with among Asian patients have failed to identify a gender-related difference [12,14,29]. In fact Liu et al. [16] found a higher incidence of cholelithiasis in males than in females below 50 years of age, but a higher incidence in females than in males in age groups above 50 years. In the present study, we did not assess the effect of gender, itself, but did assess the relative risks for pre- and post-menopausal women. Our analysis indicated that menopause is a risk factor for cholelithiasis in females. In the vacillating era of hormone replacement, the effect of estrogen might be an uncertain factor for which we were unable to control [44].

The relationship between chronic kidney disease and cholelithiasis continues to be investigated [30,45]. Chronic kidney disease may alter plasma concentrations of electrolytes, including calcium. Like bilirubin and cholesterol, calcium is a major component of gallstones. In the present study we have defined chronic kidney disease as a condition meeting the criteria for ICD-9-CM 585, 586, 588.8, or 588.9. In this context, we found no significant influence of chronic kidney disease on gallstone formation. However, since pigment gallstones predominate in Asian populations, further studies into the relationship between the renal function, parathyroid hormone, and calcium levels will be of interest.

A significant limitation of this study is that our research database did not reveal the criteria on which the diagnoses of cholelithiasis were based. Thus, it is more appropriate to describe these individuals as having symptomatic cholelithiasis as defined within the ICD-9-CM 574 guidelines, i.e., calculus of the gallbladder without mention of cholecystitis. Similarly, individuals in the control group had no history of cholelithiasis, but we are unable to rule out the possibility of asymptomatic cholelithiasis in some individuals. In addition, because of the inherent limitations of insurance data, some lifestyle risks, such as the use of ground-surface water, cannot be evaluated [46]. We were also unable to analyze the link between cholelithiasis and family history or parity in our population. Thus, further prospective studies will be required to better understand the relationship of these factors to cholelithiasis in Taiwan.

This study was designed to identify medical risk factors for cholelithiasis in the general population of Taiwan. The goal was to determine if the risk profile in the general population differed from those reported previously from studies with specific segments of the Taiwanese population, as well as from known risk factors in the West. Although no unique risk factors were identified among the population of Taiwan, this study confirms hepatitis B as a risk factor in the Taiwanese population, which is an important finding in view of the higher prevalence of hepatitis in Taiwan. The cholelithiasis-risk profile as represented in this survey of a representative population of Taiwan is similar overall to the profile that has emerged from the study of specific segments of the population, identifying obesity, hyperlipidemia, hepatitis B infection, hepatitis C infection and cirrhosis as risk factors for cholelithiasis in men and women, and menopause as an additional risk factor in females. Our study indicates that diabetes mellitus may not be an independent risk factor for cholelithiasis, and this relationship should be re-examined.

## Competing interests

The authors declare that they have no competing interests and no financial support was received.

## Authors' contributions

SCH carried out the literature research, manuscript preparation and editing. SWL performed as the guarantor of integrity of the entire study and carried out the study concepts, manuscript review and manuscript editing. CIL performed as the guarantor of integrity of the entire study and carried out statistical analysis. KFL carried out the literature research, data acquisition and study concepts. WCC carried out the manuscript review and study concepts. All authors read and approved the final manuscript.

## Pre-publication history

The pre-publication history for this paper can be accessed here:

http://www.biomedcentral.com/1471-230X/11/111/prepub
